# Astaxanthin prevents bone loss in osteoporotic rats with palmitic acid through suppressing oxidative stress

**DOI:** 10.1080/13510002.2024.2333096

**Published:** 2024-04-16

**Authors:** Zhou-Shan Tao, Xing-Jing Wu, Min Yang, Cai-Liang Shen

**Affiliations:** a Department of Orthopedics, The First Affiliated Hospital of Wannan Medical College, Yijishan Hospital, No. 2, Zhe Shan Xi Road, Wuhu, Anhui, People’s Republic of China; b Anhui Province Key Laboratory of Noncoding RNA Basic and Clinical Transformation, No. 2, Zhe Shan Xi Road, Wuhu, Anhui, People’s Republic of China; c Department of Spinal Surgery, The First Affiliated Hospital of Anhui Medical University, 218 Jixi Road, Shushan District, Hefei, Anhui, People’s Republic of China

**Keywords:** Astaxanthin, Osteoporosis, Palmitic acid, Oxidative stress, Bone metabolism

## Abstract

**Objectives:**

The study aimed to assess the role of Astaxanthin (ATX) in palmitic acid(PA) -induced bone loss in Ovariectomized(OVX) rats.

**Methods:**

In the OVX rat model, we observed that PA affects bone metabolism and accelerates bone loss. Additionally, treatment with ATX was able to suppress the deleterious effects of PA and a simultaneous decrease in serum MDA levels and an increase in SOD was observed.

**Results:**

In addition, rats treated with ATX were observed to have significantly increased bone mass and elevated activity of SIRT1 and SOD2 in bone tissue. When MC3T3-E1 and RAW264.7 cells induced osteoblast and osteoclast differentiation, the ATX intervention was able to significantly restore the restriction of osteogenic differentiation and the up-regulation of osteoclast differentiation with PA therapy. Furthermore, we confirm that PA damage to cells is caused by increased oxidative stress, and that ATX can target and modulate the activity of SIRT1 to regulate the levels of oxidative stress in cells.

**Conclusion:**

Summarizing, ATX may inhibit PA-induced bone loss through its antioxidant properties via the SIRT1 signaling pathway.

## GRAPHICAL ABSTRACT

**Figure:**
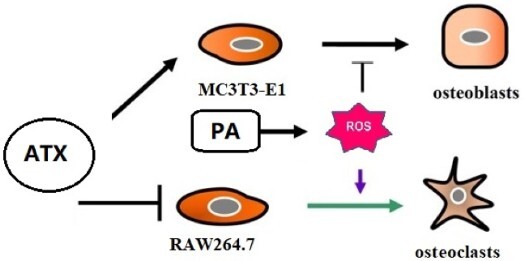

## 1 Introduction

Osteoporosis is a metabolic bone disease closely related to age, and the negative effects of the disease on people have received increasing attention as the population ages [1]. With the aging population increasing, the incidence of osteoporosis is very high and osteoporotic fracture also increases with ages [2]. Therefore, in recent years, osteoporosis has become a major public health problem in most countries, especially in postmenopausal women [3,4]. Over the past decade, substantial advances have been made in the prevention and treatment of osteoporosis, especially in drug therapy. Nevertheless, osteoporotic fracture is still associated with high disability and mortality rates in elderly patients, even with the development of technology for internal fixation and surgical technology solutions [5]. Increasing evidence has shown that hyperlipidemia increases the change in BMD and increases the risk of osteoporosis, thus the risk of certain osteoporotic complications [6]. Hyperlipidemia increases osteoclasts and affects the net loss of bone, resulting in ultimately induced bone loss and osteoporotic fracture simultaneously [7]. Additionally, obesity can induce bone apoptosis and bone adsorption by mediating multiple signaling pathways.

Current studies have shown that high circulating free fatty acids(FFA) can lead to abnormal fat metabolism and ectopic fat deposition [8], and elevated levels of FFA are one of the main characteristics of diabetes, obesity and other metabolic diseases [9]. Excessive accumulation of fatty acids in cells will adversely affect glucose homeostasis, that is, lipid toxicity [10], thus affecting various metabolic pathways and causing damage to the body. Palmitic acid (PA) is a kind of long-chain saturated fatty acid widely distributed in plants, and it is also one of the most common saturated FFAs in human body [10]. Excessive PA damages the functions of endoplasmic reticulum and mitochondria. PA can increase the levels of oxidative stress by the production of reactive oxygen species (ROS), induce apoptosis and autophagy of osteoblasts, thus affecting their differentiation and function [11,12], thus adversely affecting bone metabolism and leading to the occurrence of bone loss. Astaxanthin (ATX), a natural carotenoid widely found in algae and aquatic animals, exhibits a powerful antioxidant [13]. Because of its powerful antioxidant properties, ATX can scavenge ROS and protect the organs and cells from oxidation [14]. In addition, *in vitro* experiments showed that ATX intervention could inhibit osteoclast formation [15].

Numerous studies have confirmed the therapeutic potential and molecular mechanisms of ATX in oxidative stress. However, it is beyond our knowledge whether ATX has positive effects on PA-induced bone loss through inhibiting oxidative stress. Therefore, in this study, we explored the effect of ATX on the bone mass based on a PA-treated ovariectomized (OVX) rats model.

## 2 Materials and methods

### 2.1 Surgery and treatment

Sprague–Dawley female rats weighing 200–300 g were housed under 12 h light/dark cycles at 23°C and 60 ± 5% humidity. After purchasing, these animals were placed in a standard rearing environment and adaptively reared for 1 week. The following rats were bilaterally OVX or sham-operated as described previously [16,17]. After 3 months of normal feeding, part of the postoperative rats were killed and their femurs were extracted for further testing to confirm successful construction of animal model. Following this, all animals were randomly divided into four experimental groups: the Sham group, the OVX group, the OVX + PA group and the OVX + ATX/PA group. The rats in the OVX + PA group and the ATX/PA + OVX group were injected intraperitoneally with PA at a dose of 100 mg/kg/d (Sigma) and PA plus ATX at a dose of 50 mg/kg/d (Sigma), separately. The dose and delivery way of PA and ATX were determined according to previous experiment [18,19]. Rats were sacrificed after a 12-week treatment. Blood and bilateral femurs from all experimental rats were harvested immediately for further analysis.

### 2.2 Micro-CT scanning

At 12 weeks after treatment, the femurs harvested from each group were evaluated using a micro-CT (Skyscan 1176, Bruker, Belgium). The specific parameters of Micro-CT scanning were described as follows: 70 kVp voltage, 200 mA current, 300 ms integration time, 0.5 mm aluminum filter. For the assessment of trabecular changes, the region of analysis we chose was the growth plate extending 2 mm proximal to the femur. Data were generated for bone mineral content (BMC), bone mineral density (BMD), bone volume per total volume (BV/TV); trabecular number (Tb.N), thickness (Tb.Th), and spacing (Tb.Sp), and the mean trabecular separation (Tb.Sp) within the region of analysis as previously described [20,21].

### 2.3 Fluorescent double labeling assays

After micro-CT scanning, these femora need to undergo histological examination to assess bone mineralization rate. After being dehydrated in ethanol, these femoral samples were embedded, undecalcified, in methylmethacrylate. The handled femur were cut into 50 μm thick sections using a using a microcutter (SP1600, Solms, Germany). Then, all sections are grounded and polished to a thickness of 20 μm for analysis using a confocal laser scanning microscope (CLSM, Olympus FluoView FV1000).

### 2.4 Histological evaluation and immunohistochemical analysis

The left femurs from different groups were soaked in 10% ethylenediaminetet-raacetic acid (EDTA) for decalcification. These decalcified bone samples were cut by a standardized procedure to obtain sections. The section is required to be 5 µm thick, and the focus of the observations is on the distal femur. Then, Hematoxylin–eosin (HE) staining and Masson staining were observed and pathological pictures were obtained for analysis according to previous reports [22,23].

In addition, immunohistochemical staining was performed on the sections to obtain the changes in the activity of osteoblasts and osteoclasts in the femoral metaphysis of each group. The tissue sections were immersed in immunohistochemical blocking solution (Beyotime Biotechnology Co., Ltd., Zhejiang, China) at 37°C for 30 min to remove the endogenous catalase. Non-specific sites were blocked with bovine serum albumin (BSA) (3% for 30 min). After that, anti-Osteocalcin (OC, 1:100, Abcam, UK), anti-Tartrate-resistant acid phosphatase (TRAP, 1:100, Abcam, UK) and anti-rat secondary antibody (Beyotime Biotechnology Co., LTD, China) were dyed according to the manufacturer’s instructions. Finally, sections were observed under a fluorescence microscope, and images were collected and analyzed according to previous reports [22,23].

### 2.5 Immunofluorescence staining

Bone tissue sections were acquired after decalcification, embedding and slicing. Antigen retrieval was performed by incubation with citrate buffer (pH 6.0) at 95°C for 15 min. Afterwards, the sections were incubated with 10% normal serum for 1 h at room temperature. Expression of superoxide dismutase 2 (SOD2, Abcam, 1:200) and nad-dependent deacetylase sirtuin-1 (SIRT1, Abcam, 1:200) were investigated using a rabbit polyclonal primary antibody, followed by fluorescent (Jackson Immuno Research, 415-605-166, 1:500; 315-605-003, 1:250), before mounting with Vectashield containing DAPI (Vector Laboratories, Burlingame, CA). Finally, a confocal microscope (FLUOVIEW FV300, Olympus) was used for image acquisition and analysis.

### 2.6 Measurement of oxidative stress and markers of bone turnover in serum

Bone turnover markers including Alkaline phosphatase(ALP), osteocalcin (OC), C-terminal telopeptide of type 1 collagen (CTX), and Tartrate-resistant alkaline phosphatase 5b (TRAP-5b) were analyzed using a commercially available ELISA kit (Takara, Co, Ltd.). Malondialdehyde (MDA) and superoxide dismutase (SOD) were detected in the serum by using the MDA assay kit and SOD activity assay kit respectively according to the manufacturer s specifications.

### 2.7 Cell experiment

#### 2.7.1 MC3TE-E1 cell and RAW264.7 cell experiments

The murine osteoblast cell line MC3T3-E1 (Shanghai Cell Bank) was used as *in vitro* model. Mouse macrophage RAW264.7 cells (Shanghai Bangjing Industrial Co., Ltd. China) were used to test the inductive effect of PA on osteoclastogenesis. Dulbecco’s Modified Eagle’s Medium (DMEM, Gibco Inc.) was formulated as the cell culture medium for RAW264.7 and MC3T3-E1 cells. Initially MC3TE-E1 Cells and RAW264.7 cells were cultured with different environment with different intervention as follows: normal medium(Con); Palmitic acid treatment group(0.4 mmol/L PA); Palmitic acid(0.4 mmol/L) plus ATX(50µmol/L) treatment group(ATX/PA) and Palmitic acid/ATX + EX527(a SIRT1 inhibitor, 10 μM; Selleck Chemicals, Houston, TX, USA) treatment (ATX/PA + EX527). Dosages used for EX527 and ATX were chosen according to previous studies [24,25].

#### 2.7.2 Cell proliferation assay

MC3T3-E1 cells were cultured in 24-well plates at a density of 5 × 10^4^ cells per well and then incubated with different concentrations PA overnight. The effects of different concentrations (0, 0.2, 0.4, 0.6, 0.8, 1 mmol/L) of PA and ATX (50 µmol/L) on cell proliferation activity at different times (6, 12, 24 h) were detected by CCK-8 kit. Then, 100 μL fresh medium containing 10 μL CCK-8 solution was added to each well. Afterwards, cells were incubated for 2 h in 37℃ incubator. At 450 nm, the microplate reader measured absorbance as previously described [26,27].

#### 2.7.3 Induction and evaluation of MC3TE-E1 osteogenesis

MC3T3-E1 cells were cultured in 24-well plates at a density of 5 × 10^4^ cells per well and then incubated with PA and ATX. When MC3T3-E1s were grown to 70% confluency, the medium was changed to an osteoinductive medium (10 mM β-glycerophosphate and 280 μM ascorbic acid) to induce osteogenesis. After 14 days of osteogenic induction, ALP staining kit (Beyotime, Shanghai, China) was used to stain the cells in the medium to determine the expression of ALP. Cultured under the same conditions for 21 days, alizarin red (alizarin red S; Beyotime, China) was used to stain the cells in the medium to determine the ability of cells to form calcified nodules as previously described [28,29].

#### 2.7.4 Evaluation of oxidative stress

After 48 h of intervention, the changes in ROS levels of MC3TE-E1 cells following the intervention were evaluated using 2′,7′-dichlorofluorescein diacetate (DCFH-DA; Sigma, St. Louis, MO) staining and MitoSOX™ Red mitochondrial superoxide indicator (MitoSOX™; Sigma, St. Louis, MO) staining.

To confirm the changes in mitochondrial membrane potential, we use co-stained with tetramethylrhodamine methyl ester (TMRM, 100 nM, Life Technologies) and Mitotracker Green (Mitogreen, 100 nM, Life Technologies) to observe and evaluate the state of the mitochondrial oxidative stress in treated cells. After 48 h of intervention, TMRM and Mitogreen staining were performed as previously described [30]. The results of TMRM (excitation wavelengths = 543 nm) and Mitogreen (excitation wavelengths = 488 nm) were captured using Laser confocal microscope. The quantification of mitochondrial membrane potential was determined using Image J software.

Immunofluorescence staining was used to quantitatively detect and evaluate the expression of SIRT1 and SOD2 in MC3T3-E1 after different interventions to understand the changes of cellular oxidative stress as stated above. The levels of Malondialdehyde (MDA) and total SOD activity in the cells were measured by a malondialdehyde Detection Assay kit (APPLYGEN) and Superoxide Dismutase Activity Assay Kit (Abcam), respectively.

#### 2.7.5 Osteoclast induction of RAW264.7 cells

RAW264.7 cells were seeded in 96-well plates (1 × 10^4^ cells/well) and grown in DMEM (Gibco-BRL, Grand Island, NY, USA). When the cells reached 80% confluence, 50 ng/mL of RANKL was added to the medium to induce osteoclast differentiation. Afterwards, the cell culture medium was added with PA and ATX and cultured for 7 days. RAW264.7 cells were then stained for tartrate-resistant acid phosphatase (TRAP) as described previously [31].

### 2.8 Statistical analysis

All the quantitative data used in this study were analyzed. One-way analysis of variance (One-way ANOVA) was used for multiple group comparisons followed by Tukey’s *post hoc* test and expressed as the means ± SD with *n* ≥ 3. Significance was measured at the following thresholds: *P < 0.05.

## 3 Results

### 3.1 ATX inhibits the negative effects on bone mass and BMD in osteoporosis rats

**Figure 1.**
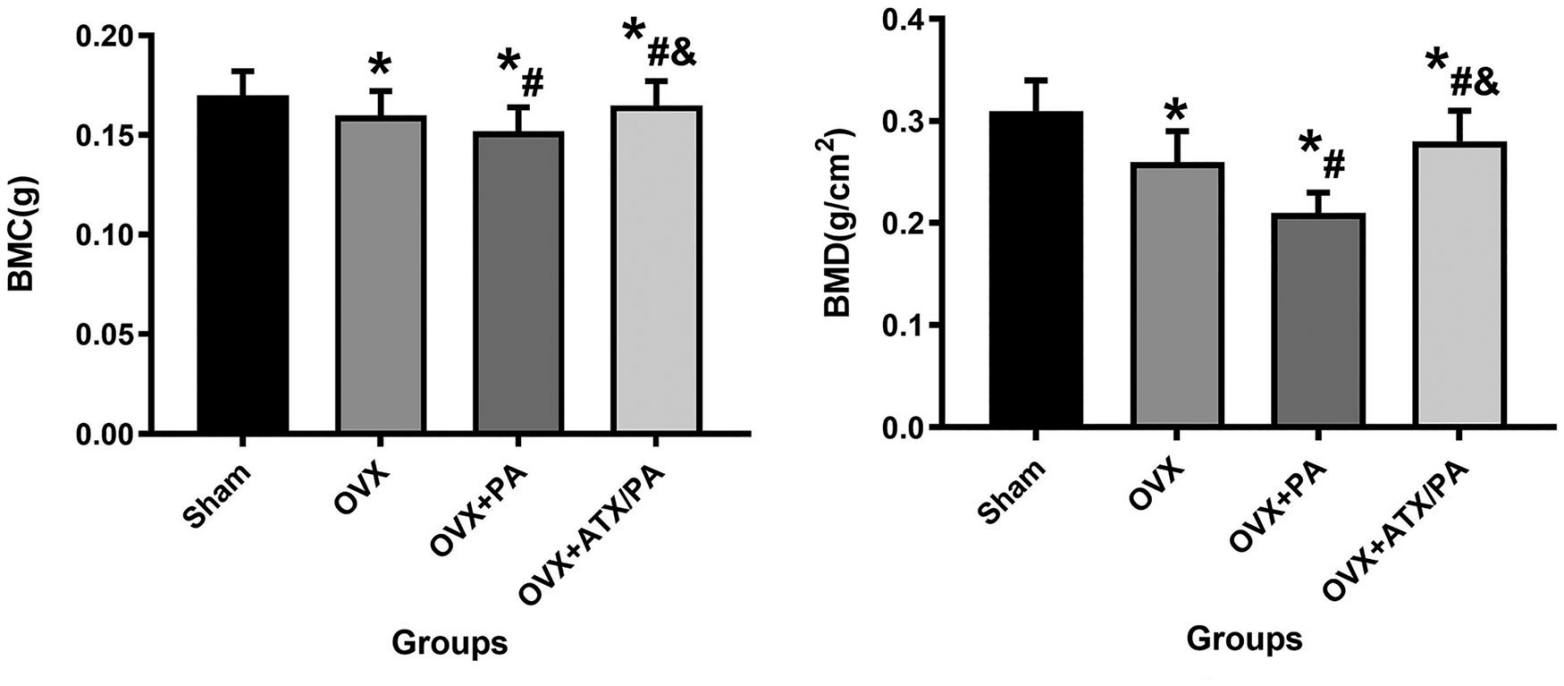
ATX significantly inhibited the reduction of BMD and BMC in PA-treated OVX rats by Micro-CT. *vs Sham group (*P *< 0.05); **^#^**vs OVX group (*P *< 0.05); **^&^**vs OVX + PA group (*P *< 0.05).

**Figure 2.**
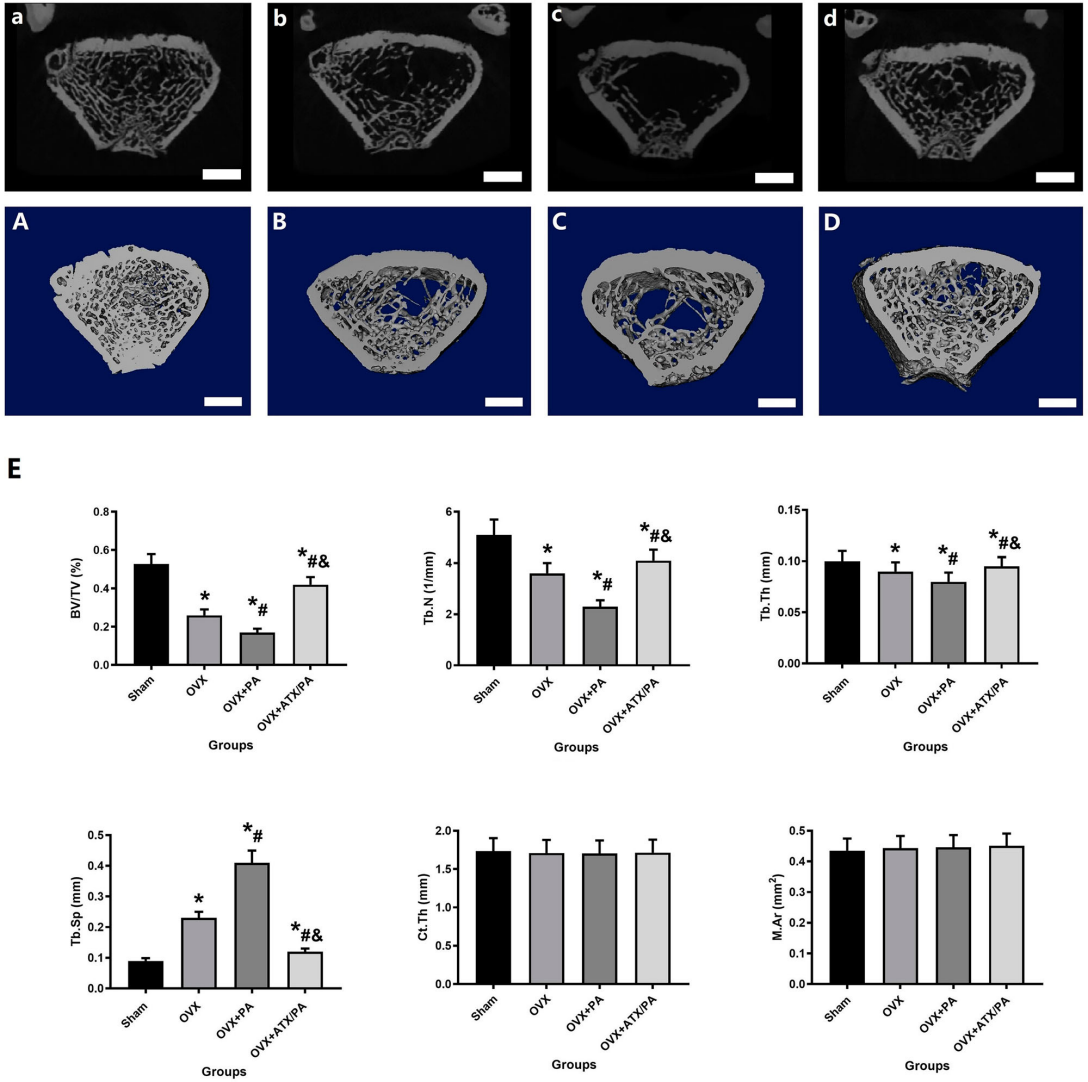
Micro-CT 2D and 3D images and evaluation of the microscopic parameters of the trabeculae of treated OVX rats. Micro-CT 2D and 3D images of rats belonging to Sham (A, a), OVX (B, b), OVX + PA (C, c), and OVX + ATX/PA (D, d) groups (scale bar = 1 mm). E. The trabecular parameters of the distal femur include BV/TV, Tb.N, Tb.Sp, Tb.Th, Conn.D, and BMD. *vs Sham group (*P *< 0.05); **^#^**vs OVX group (*P *< 0.05); **^&^**vs OVX + PA group (*P *< 0.05).

**Figure 3.**
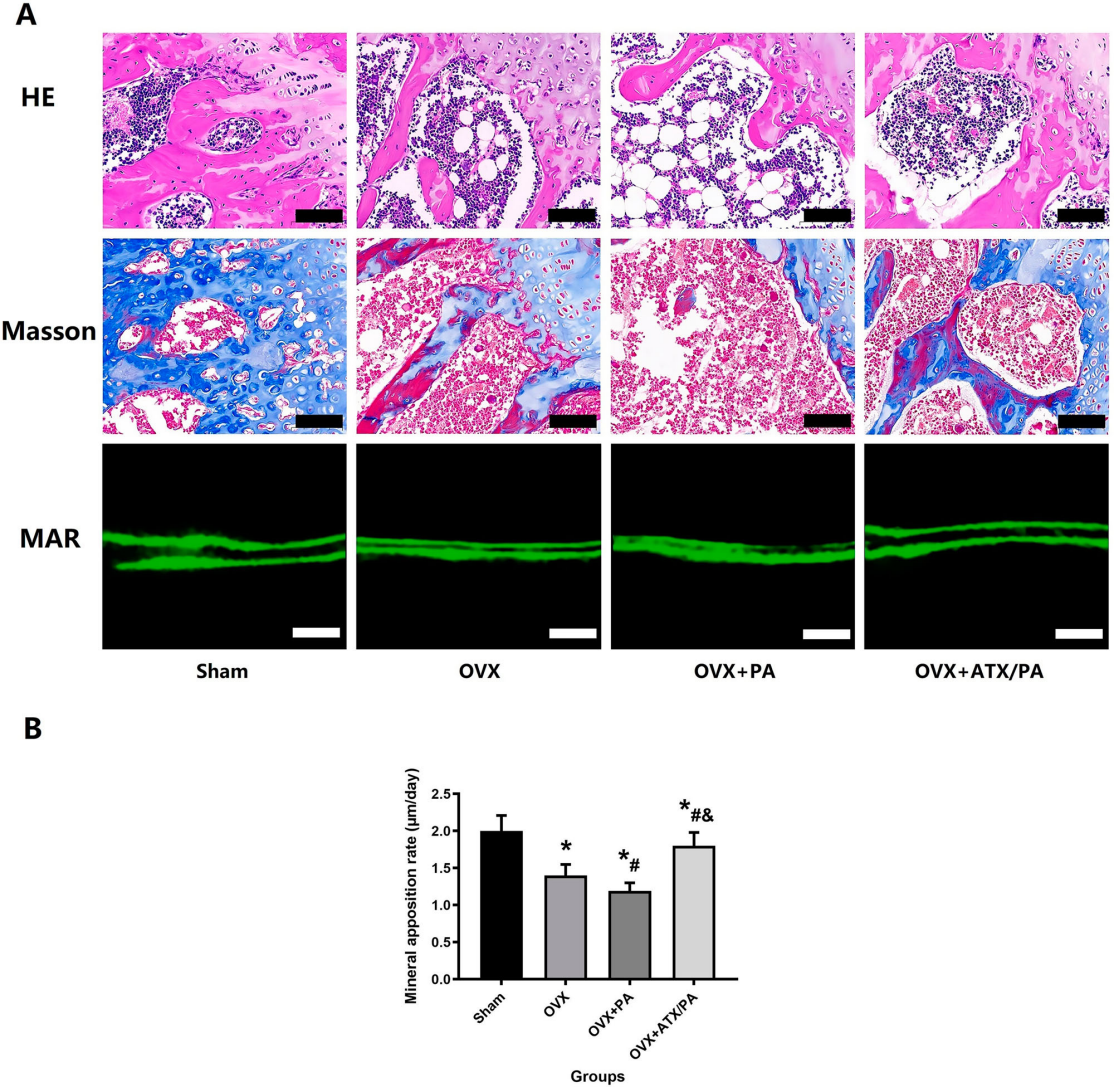
Treatment with ATX prevented bone loss and increased mineral apposition rate in OVX rats treated with PA. (A) Representative images of HE staining and Masson’s staining of the metaphyseal tissue of the distal femur (Magnification, ×20) and mineral apposition rate (Magnification, ×200). (B) Quantitative analysis of MAR. *vs Sham group (*P *< 0.05); **^#^**vs OVX group (*P *< 0.05); **^&^**vs OVX + PA group (*P *< 0.05).

To verify the protective effect of ATX on PA intervention on bone mass in the distal femur of osteoporotic rats, micro-CT and HE were performed to detect changes in BMD, BMC, and microstructure in the trabecular bone of the distal femur. BMD and BMC were significantly lower in the PA + OVX group compared to the OVX group. However, after the ATX intervention, the BMD and BMC values increased significantly (*P* < 0.05, Figure 1). Distal femur trabecular bone parameters were also analyzed using Micro-CT (Figure 2). In the distal femur, micro-CT evaluation revealed that the PA + OVX rats had lower BV/TV, Tb.Th, Tb.N, as well as higher Tb.Sp compared to the OVX group (*P* < 0.05). However, after the ATX intervention, these bone trabecular parameters showed significant improvement. (*P* < 0.05). HE and Masson staining of the sagittal plane of the distal femur revealed that the OVX group showed more severe bone trabecular loss after PA treatment, replaced by large amounts of fatty cells. However, these poor bone trabeculae were recovered by ATX treatment (Figure 3). Compared with OVX group, the poor bone mass in OVX + PA group was significantly reduced by Micro-CT evaluate, HE and masson stained section, with a great improvement after ATX treatment (Figure 3).

To further observe the effect of ATX on osteogenic activity in PA-treated rats, bone Mineral apposition rate (MAR) was labeled and analyzed. PA adversely affected MAR in aged rats by fluorescence double labeling, and ATX treatment improved MAR (Figure 3B).

### 3.2 ATX regulate the activity of osteoblast and osteoclast in osteoporotic rats

**Figure 4.**
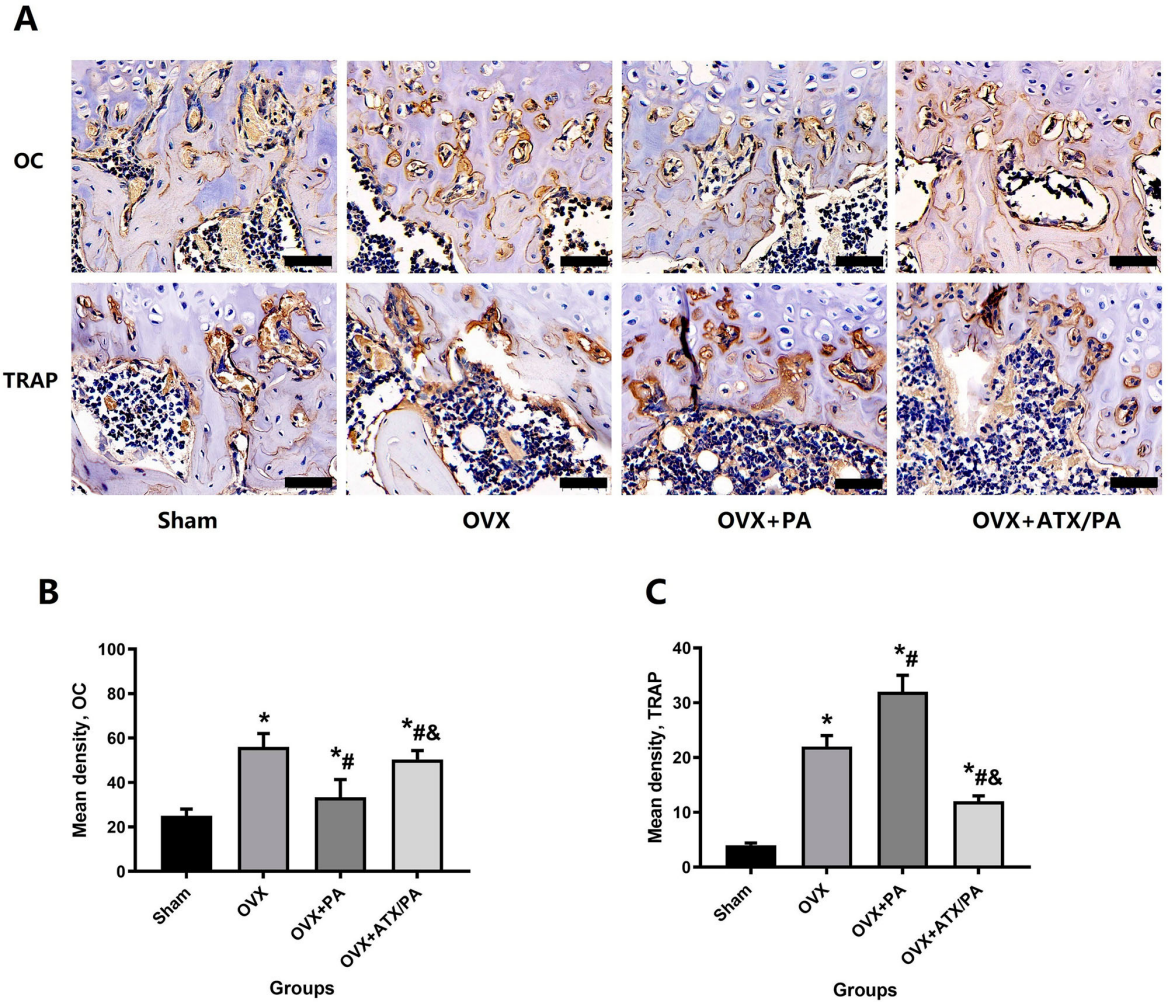
Systemic administration of ATX modulates osteoblast and osteoclast activity in OVX rats treated with PA. (A) Representative images were analyzed by immunohistochemistry for OC and TRAP. (B) Quantitative analysis of OC and TRAP (magnification, ×63). *vs Sham group (*P *< 0.05); **^#^**vs OVX group (*P *< 0.05); **^&^**vs OVX + PA group (*P *< 0.05).

**Figure 5.**
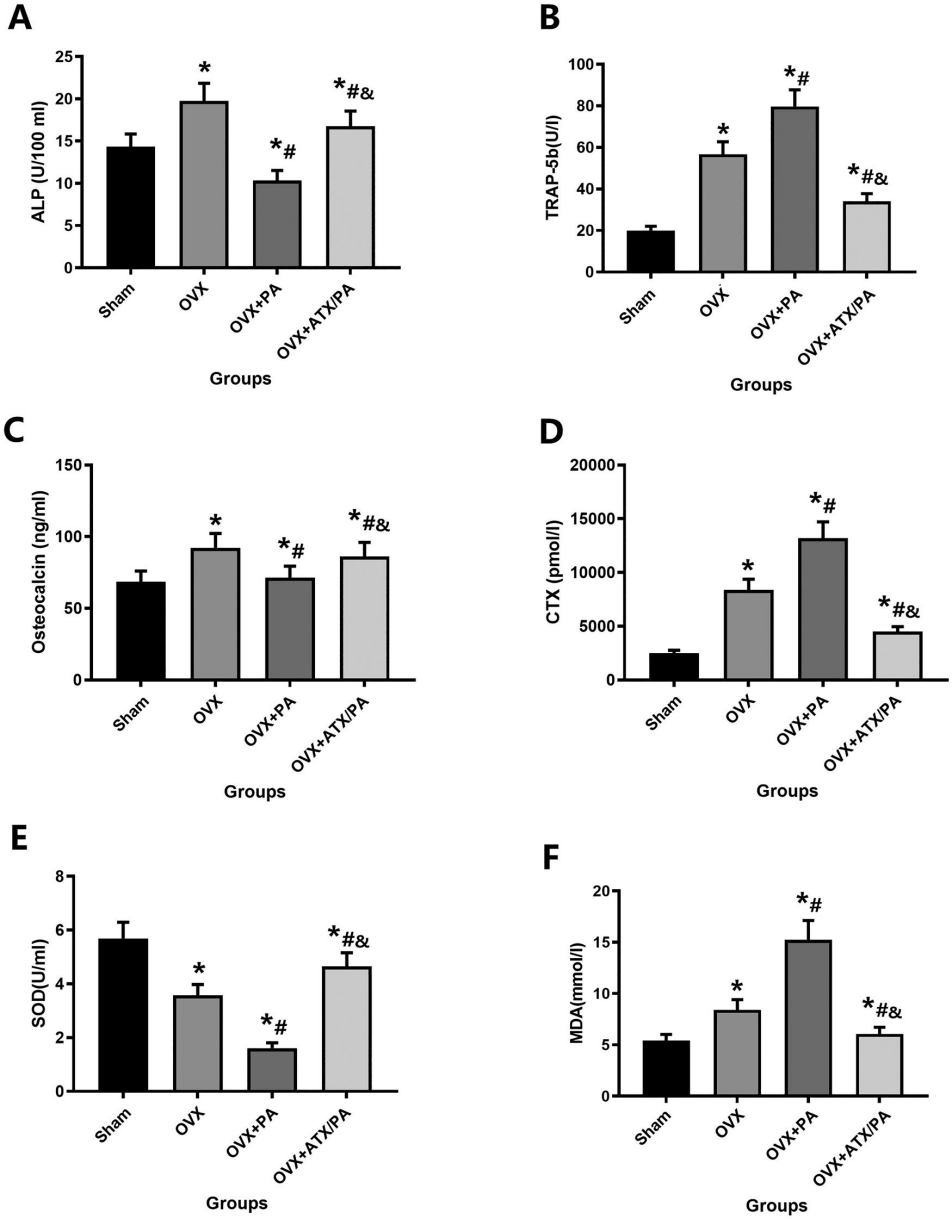
Systemic administration of ATX modulates levels of specific indicators, including bone metabolism and oxidative stress, in OVX rats treated with PA. Serum levels of ALP (A) and OC (C) were evaluated as markers of bone formation. Serum levels of the bone resorption markers TRAP-5b (B) and CTX (D). Levels of the oxidative stress markers SOD (E) and MDA (F). *vs Sham group (*P *< 0.05); **^#^**vs OVX group (*P *< 0.05); **^&^**vs OVX + PA group (*P *< 0.05).

Since the activity of osteoblasts and osteoclasts plays an influential role in the pathogenesis and development of osteoporosis, we also used immunohistochemical methods to label TRAP and OC for bone reconstruction analysis (Figure 4A). Our results showed that PA treatment significantly reduced OC expression and increased TRAP expression compared with the OVX group (*P* < 0.05). Besides, ATX-treated rats significantly increased OC expression and reduced TRAP expression in contrast to the PA + OVX group (*P* < 0.05, Figure 4B, C). In addition, we detected changes in various specific indexes of bone metabolism in serum in each group, including OC, ALP, CTX, and TRAP-5b. Our results showed that PA treatment significantly reduced OC and ALP levels and increased TRAP-5b and CTX levels compared with the OVX group (*P* < 0.05). Besides, ATX-treated rats significantly increased OC and ALP levels and reduced TRAP-5b and CTX levels in contrast to the FAC group (*P* < 0.05, Figure 5A–D).

### 3.3 ATX reduces oxidative stress in osteoporotic rats

Serum marker levels including MDA and SOD in our study were used to indicate for reflection oxidative stress levels in rats. Our results showed that PA treatment significantly reduced SOD levels and increased MDA levels compared with the OVX group (*P* < 0.05). Besides, ATX-treated rats significantly increased SOD levels and reduced MDA levels in contrast to the OVX group (*P* < 0.05，Figure 5E,F).

**Figure 6.**
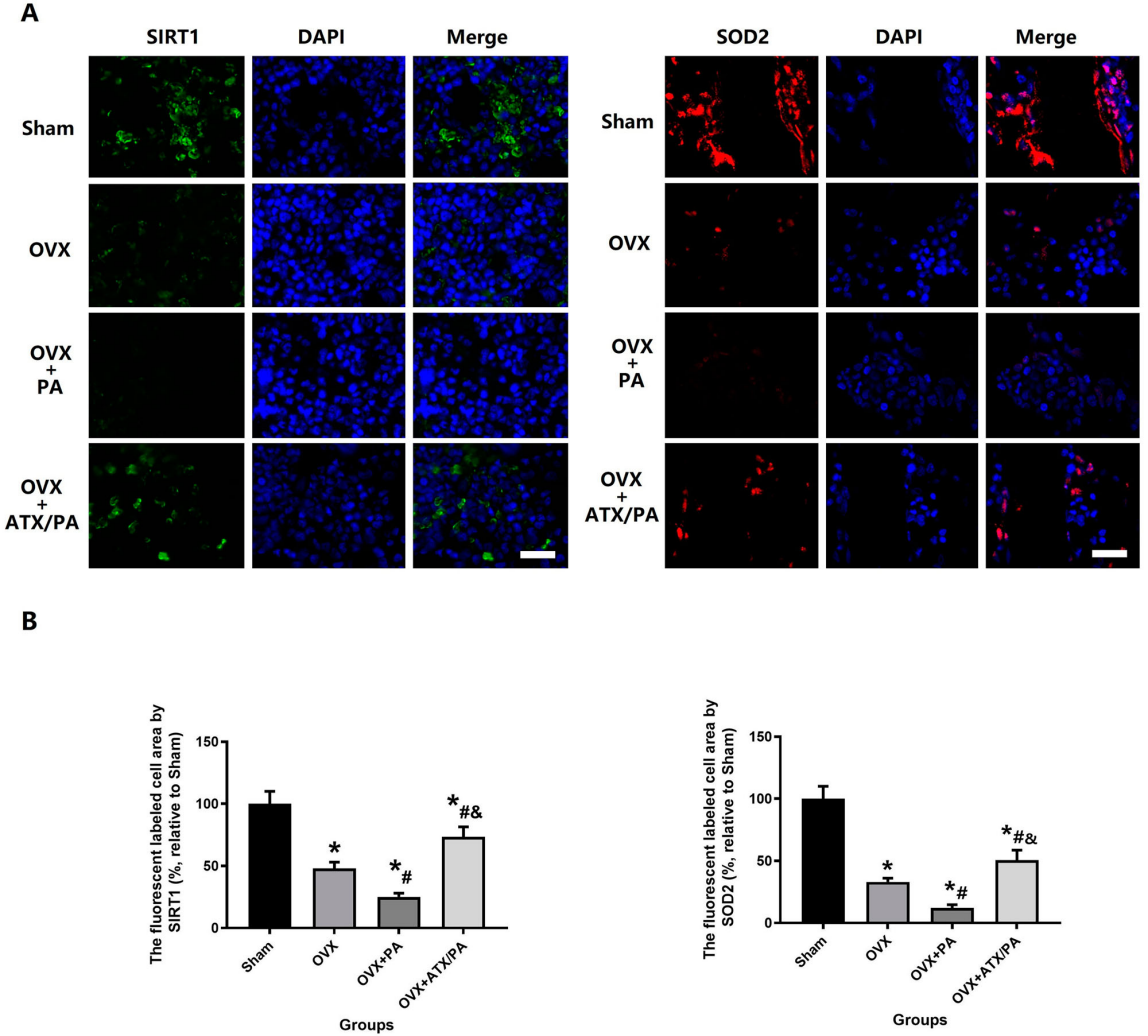
ATX increase the expression of SIRT1 and SDO2 in OVX rats treated with PA. (A) Representative images of the expression of SIRT1 in the metaphyseal tissue of the distal femur (scale bar = 25 µm). (B) Quantitative analysis of the expression of SIRT1 and SDO2. *vs Sham group (*P *< 0.05); **^#^**vs OVX group (*P *< 0.05); **^&^**vs OVX + PA group (*P *< 0.05).

Previous studies have shown that the biological process of oxidative stress is also regulated by SIRT1 and SOD2 [26,27,32]. To determine the effects of ATX on SIRT1 and SOD2 in PA-treated rats, we also used immunofluorescence methods to label for analysis (Figure 6A). Our results showed that PA treatment significantly reduced the expression of SIRT1 and SOD2 compared with the OVX group (*P* < 0.05). Besides, ATX-treated rats significantly increased expression of SIRT1 and SOD2 in contrast to the PA + OVX group (*P* < 0.05, Figure 6B).

### 3.4 ATX improves the proliferation of MC3T3-E1 cells

**Figure 7.**
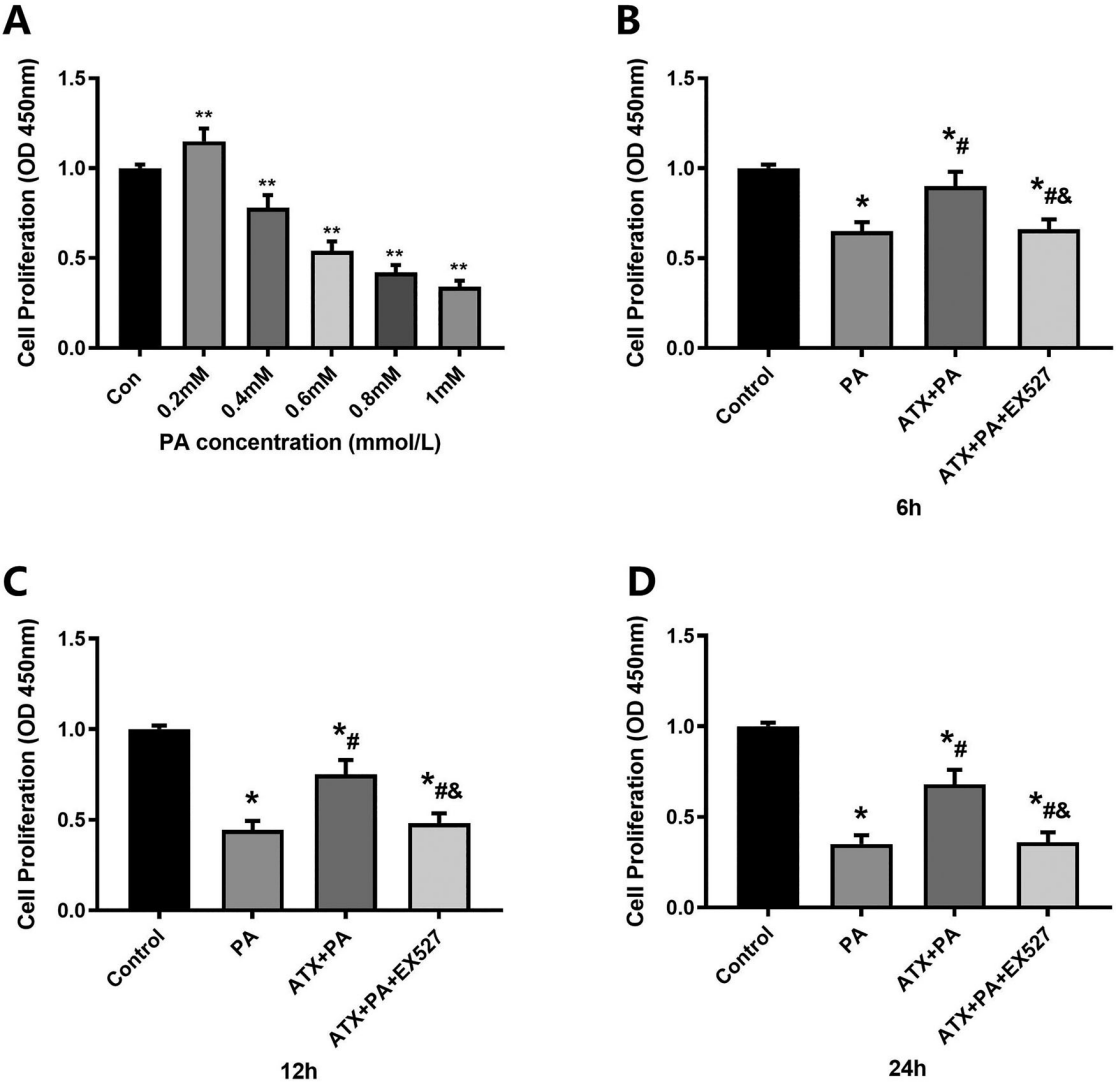
Effect of ATX on the proliferation rate of MC3T3-E1 cells treated with PA. (A) The effect of PA on cell proliferation rate at different concentrations was detected by CCK-8 for 24 h; **vs Con group (*P *< 0.01). (B–D) The effect of ATX on the rate of cell proliferation was detected by CCK-8 at a concentration of 20 uM PA for 6, 12 and 24 h. *vs Control group (*P *< 0.05); **^#^**vs PA group (*P *< 0.05); **^&^**vs ATX + PA group (*P *< 0.05).

To detect the effect of different concentrations of ATX and PA on cell proliferation rate, different concentrations of PA (0.2, 0.4, 0.6, 0.8, 1.0 mM) were applied to cells for 24 h in this experiment, and the cell proliferation rate was detected by CCK-8 assay. As shown in Figure 7A, the cell proliferation rate shows a downward trend when the PA concentration ranges from 0.2 to 0.4 mM. When the concentration of PA was fixed at 0.4 mM, the effects of different action times (6, 12, 24 h) on the proliferation rate of MC3T3-E1 were investigated. As shown in Figure 7B–D, CCK-8 results showed that the cell proliferation rate of PA was reduced at 6, 12 and 24 h after PA treatment, while ATX treatment could significantly inhibit the adverse effects of PA, and the above effects of ATX were interfered by EX527.

### 3.5 ATX protect the damage of PA on the osteogenic ability of MC3T3-E1

**Figure 8.**
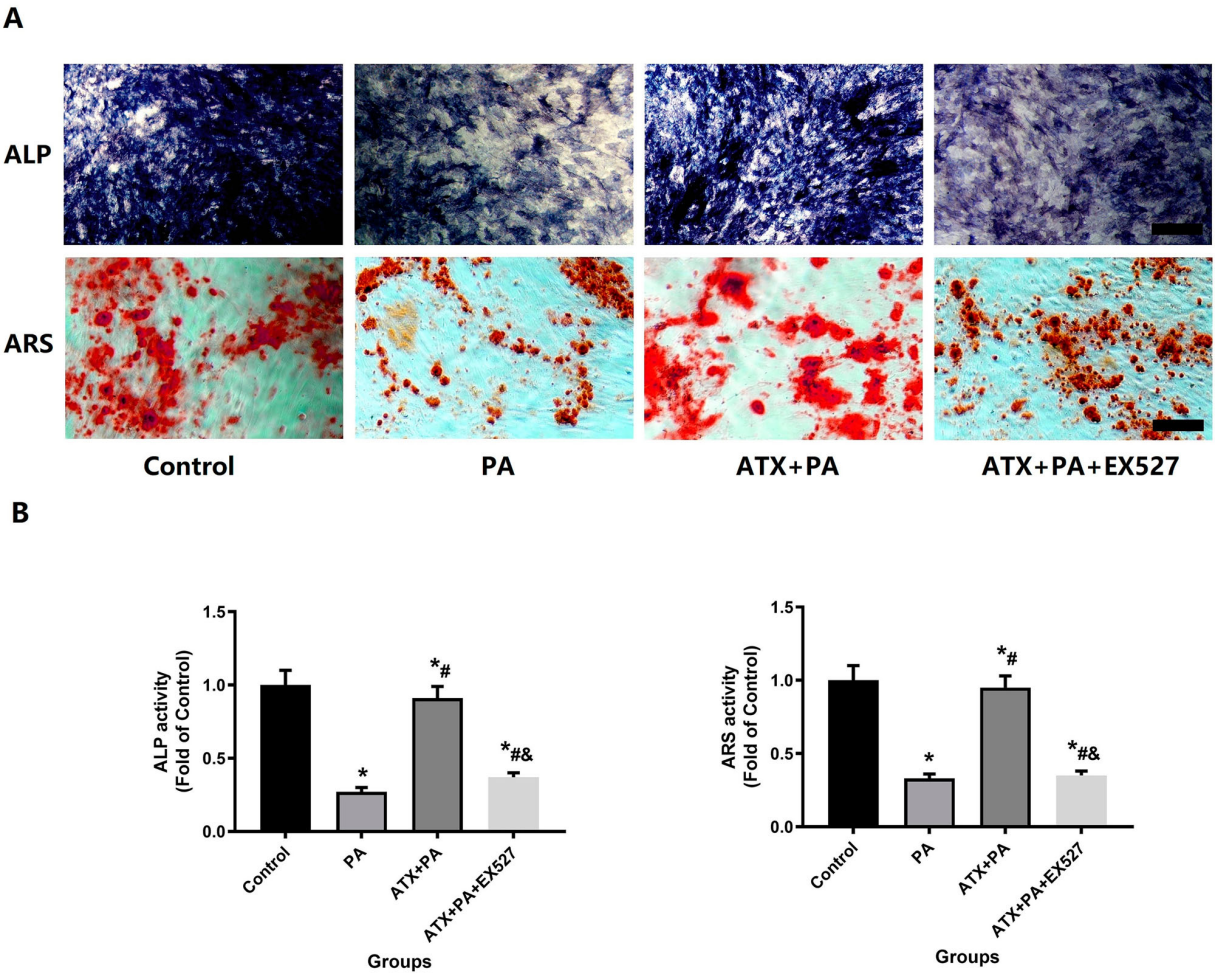
Effect of ATX intervention on MC3T3-E1 osteogenesis by alkaline phosphatase staining and calcified nodule formation by alizarin red staining with PA treatment. (A) After the addition of ATX, the expression of alkaline phosphatase and the formation of calcified nodules increased significantly (scale bar = 50 µm). (B) Quantitative analysis of positive areas of alkaline phosphatase staining and positive areas of alizarin staining. *vs Control group (*P *< 0.05); **^#^**vs PA group (*P *< 0.05); **^&^**vs ATX + PA group (*P *< 0.05).

Based on the results of ALP staining and alizarin staining(Figure 8A) in MC3T3-E1 was significantly reduced after treatment with PA, mineralization was significantly reduced, and calcium nodule formation was reduced, while ATX treatment could significantly inhibit the adverse effects of PA, and the above effects of ATX were interfered by EX527 (Figure 8B).

**Figure 9.**
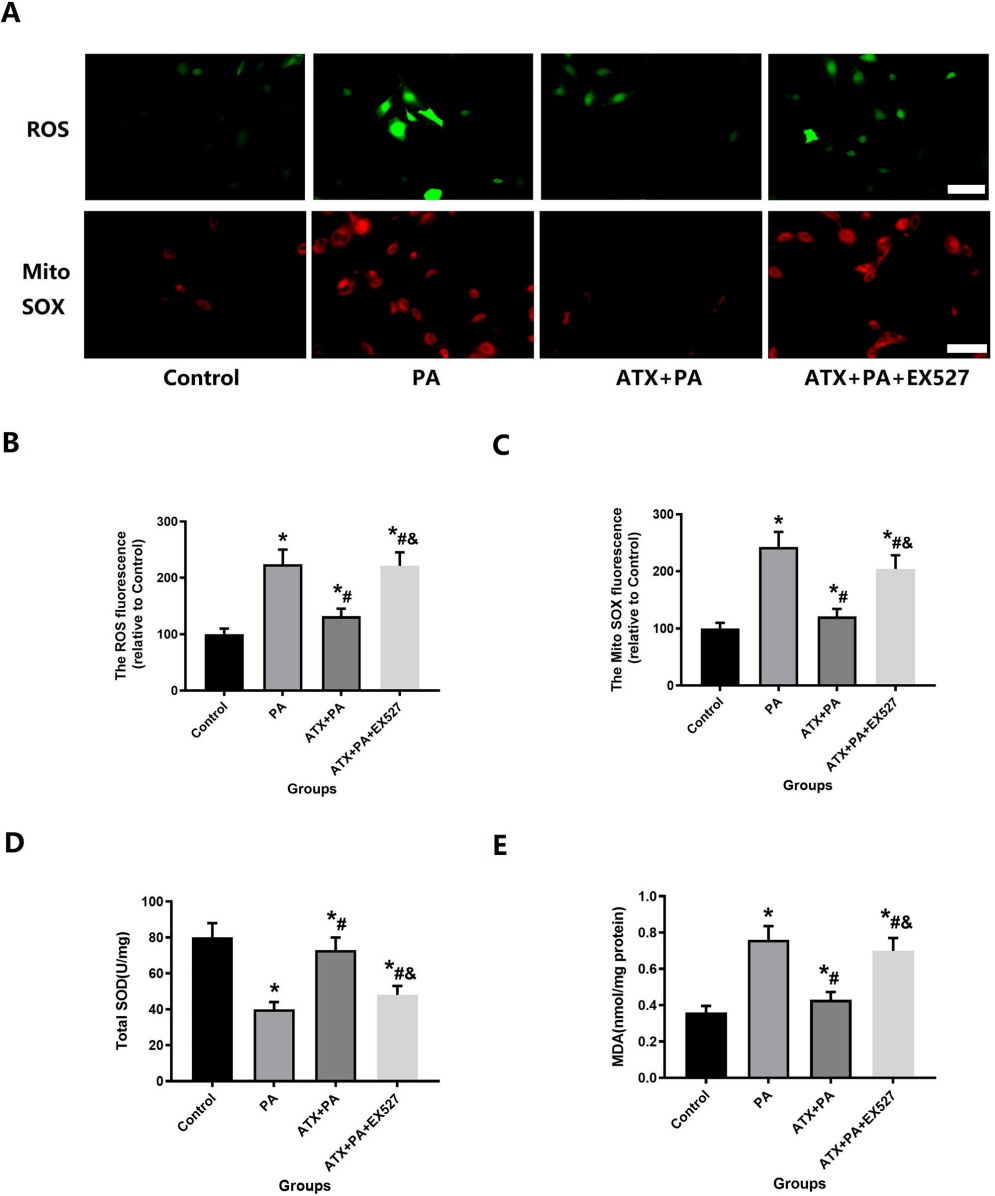
ATX treatment can reduce the elevated levels of MC3T3-E1 oxidative stress caused by PA intervention. (A) ATX treatment can reduce the elevated levels of MC3T3-E1 ROS and Mito SOX caused by PA intervention(scale bar = 25 µm); (B,C) quantitative analysis of positive fluorescence intensity of ROS and Mito SOX staining MC3T3-E1; (D, E) SOD and MDA levels were significantly changed after ATX and PA intervention. * vs Control group (*P *< 0.05); **^#^**vs PA group (*P *< 0.05); **^&^**vs ATX + PA group (*P *< 0.05).

**Figure 10.**
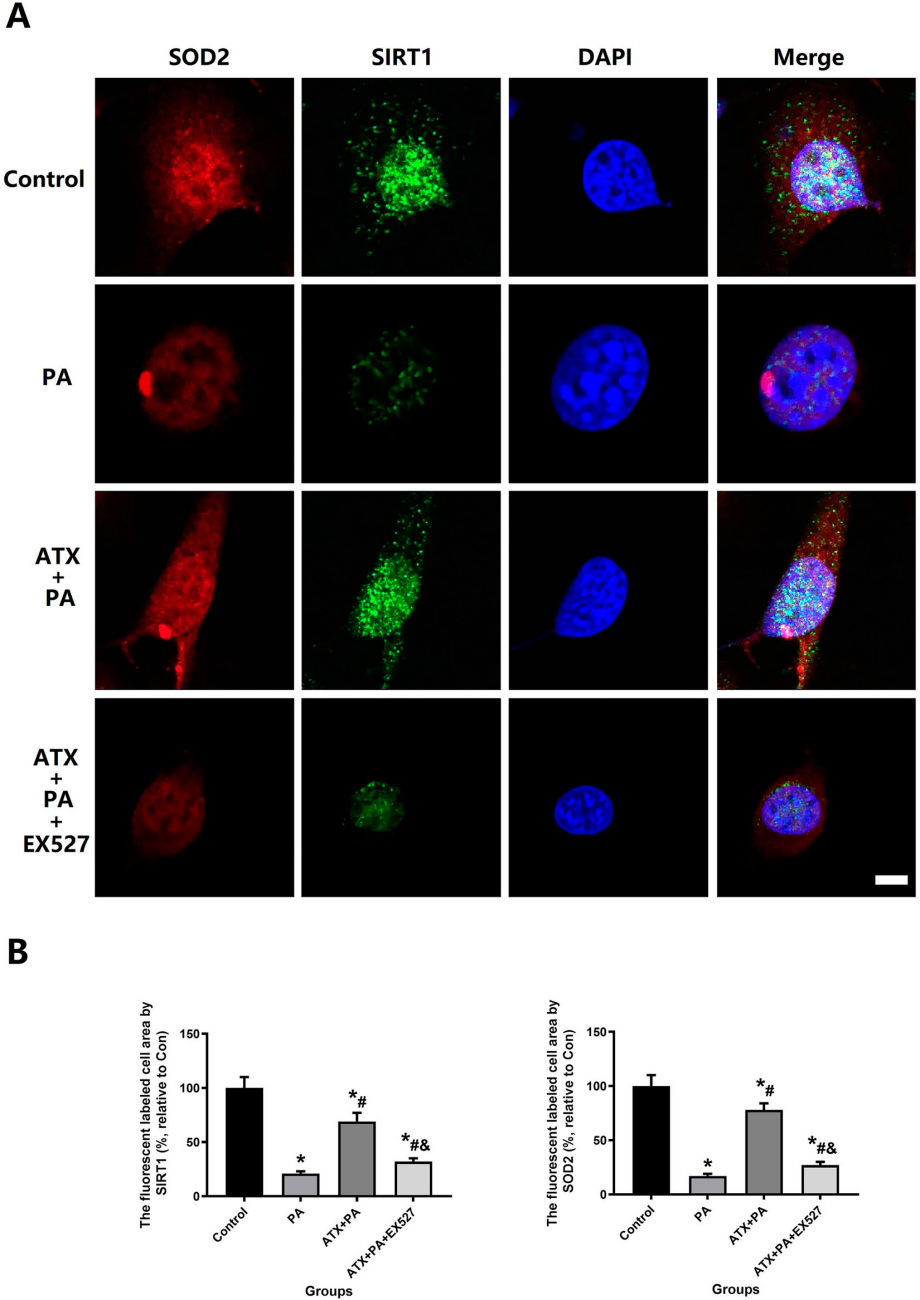
ATX intervention increased MC3TE-E1 resistance to oxidative stress and was analyzed by SIRT1 and SOD2 expression. (A) SIRT1 and SOD2 were observed at high magnification to assess the ability of ATX to significantly improve the resist oxidative stress caused by PA intervention in MC3TE-E1 (Scale bar = 25 µm); (B) quantitative analysis of changes in SIRT1 and SOD2 expression in MC3TE-E1 after ATX and PA interventions. *vs Control group (*P *< 0.05); ^#^vs PA group (*P *< 0.05); ^&^vs ATX + PA group (*P *< 0.05).

At present, a large number of studies have confirmed that the adverse effects of PA are closely related to the oxidative stress damage [11,12], so we further examined the oxidative stress state of MC3T3-E1. First, we detected ROS and Mito SOX production in different groups of MC3T3-E1 after treatment with immunofluorescence stain (Figure 9A). The results clearly and intuitively indicate that ROS and Mito SOX production is abundant after the intervention of PA and that ATX is able to suppress ROS and Mito SOX production (Figure 9B,C). At the same time, the SOD and MDA levels of MC3T3-E1 also changed significantly after the intervention of PA and ATX (Figure 9D, E). In addition, ATX therapy can markedly enhance the intracellular expression of SOD2 and SIRT1 of PA-treated osteoblasts (Figure 10).

**Figure 11.**
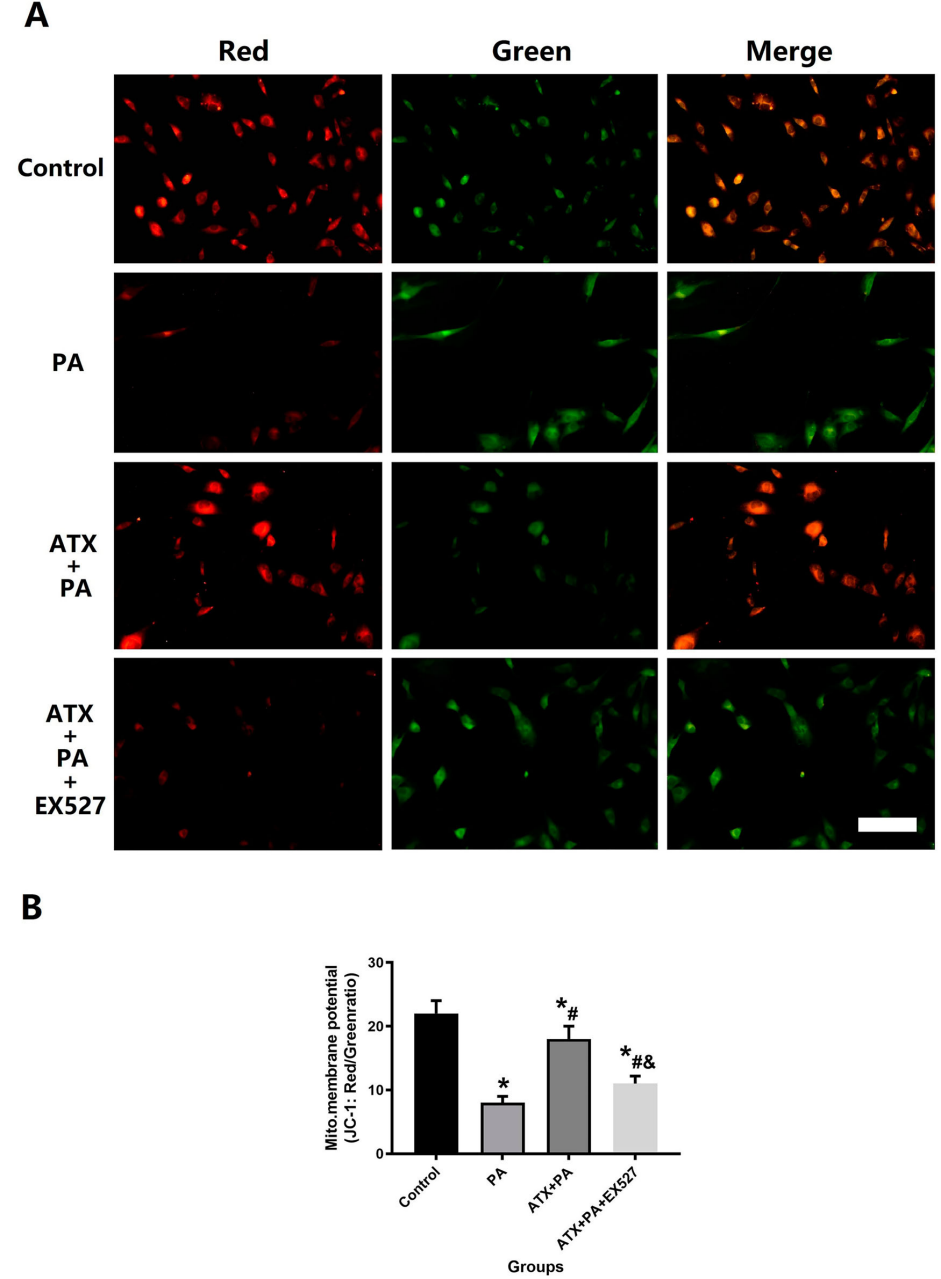
ATX intervention promotes recovery of mitochondrial membrane potential activity in the presence of PA by JC-1 staining. (A) JC-1 stainings were observed at high magnification to assess the ability of ATX to significantly improve the recovery of mitochondrial membrane potential in PA-treated MC3TE-E1 (scale bar = 25 µm); (B) quantitative analysis of changes in mitochondrial membrane potential in MC3TE-E1 treated with PA after ATX intervention. *vs Control group (*P *< 0.05); ^#^vs PA group (*P *< 0.05); ^&^vs ATX + PA group (*P *< 0.05).

We also observed changes in the mitochondrial membrane potential of MC3T3-E1 in each group through JC-1 detection and analysis to understand the state of cellular oxidative stress at the organelle level (Figure 11A). The results of JC-1 suggest that the PA can cause damage to the mitochondrial membrane potential of MC3T3-E1 and that ATX can resist the damage of the PA on the mitochondrial membrane potential (Figure 11B). Interestingly, all of the above variations can be disrupted by EX527.

### 3.6 ATX inhibition the increased osteoclast capacity in RAW264.7 cells under PA intervention

**Figure 12.**
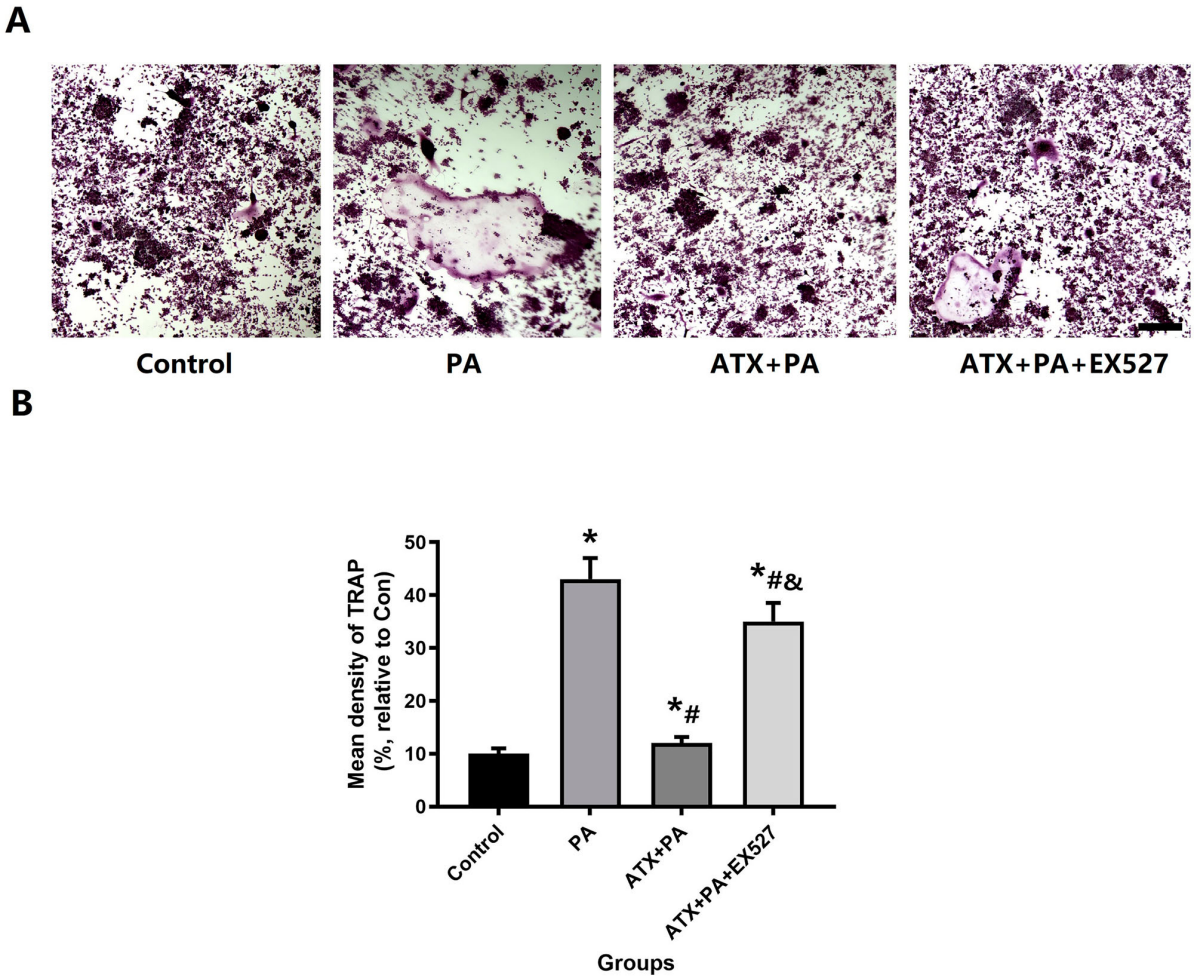
The increased osteoclast capacity in RAW264.7 cell treated by PA was disturbed by ATX treatment. (A) After adding ATX, the higher expression of TRAP decreased significantly by PA (scale bar = 50 µm); (B) for the quantitative analysis of TRAP staining positive areas. *vs Control group (*P *< 0.05); ^#^vs PA group (*P *< 0.05); ^&^vs ATX + PA group (*P *< 0.05).

Based on the results of TRAP staining (Figure 12A) and quantitative analysis, the expression of TRAP in RAW264.7 was significantly increased after treatment with PA. While ATX treatment could significantly inhibit the positive effects of PA, and the above effects of ATX were interfered by EX527.

## 4 Discussion

Hyperlipidemia has become one of the most common metabolic diseases in the world that seriously harm human health, but its possible impact on bone health has not yet attracted sufficiently attention from clinicians and society. Recent study has demonstrated the adverse effects of a high-fat diet on bone loss [33]. It has been proposed that PA causes endoplasmic reticulum stress by activating a type of apoptosis-induced factor-mediated apoptosis via the mitochondrial pathway [34]. Studies have found that PA is one of the most common saturated fatty acids in bone marrow of women with osteoporosis, suggesting that PA may produce lipid toxicity to bone metabolism [35]. Hence, we hypothesize that PA is not friendly to bone metabolism and therefore leads to impaired bone remodeling and bone loss. First, we visually evaluated the effect of PA on bone mass and microstructure of bone trabecula in the femur of an osteoporotic animal model. With respect to imaging, histological, and biomechanical data, our results suggest that PA reduces bone strength and accelerated bone loss under the conditions of an animal model of osteoporosis. Interestingly, in the presence of ATX, the adverse effects of PA are disturbed. In other words, ATX prevents bone strength loss and bone loss from the harmful effects of PA.

Since bone repair is closely related to the activity of osteoblasts and osteoclasts [36], it is important to conduct *in vitro* experiments to explore the effects of different intervention factors on osteoblasts and/or osteoclasts. In addition, the impaired surface bone formation and poor bone strength in this study may be related to decreased osteo blast activity or/and increased osteoclast function. Therefore, this study also explores the effects of ATX on differentiation and function in MC3TE-E1 cells and RAW264.7 cell treatment with PA. When MC3T3-E1 induced osteoblast differentiation, PA intervention showed significant restriction of osteogenic differentiation, as well as reduced expression of ALP and calcified nodule formation. However, the negative effect of PA on osteoblast differentiation was disturbed after the addition of ATX. These results suggest an adverse effect of experimental doses of PA on bone formation, which ATX can remove. When the differentiation of RAW264.7 cells into osteoclasts was induced, PA intervention showed obvious up-regulation of osteoclast differentiation. Significantly, the improvement in differentiation of RAW264.7 cells induced by PA was disrupted by the addition of ATX. These *in vitro* results preliminarily suggested that the harmful effect of PA on bone metabolism and bone mass are related to inhibition of osteoblast activity/function and stimulation of osteoclast activity. Interestingly, ATX inhibits the adverse effects of PA to protect bone metabolism and inhibit bone loss.

A growing number of research has come to the consensus that oxidative stress not only impairs bone strength and quality but also inhibits bone marrow-derived stem cells from differentiating into osteoblast cells [37]. It has been shown that ROS production and excess accumulation are crucial factors in inhibiting osteogenic differentiation and increasing osteoclastic activity [38]. Our animal experiments have observed that PA significantly upregulates oxidative stress levels in osteoporotic rats, as indicated by a decrease in SOD and an increase in MDA levels, which is confirmed by changes in the fluorescence intensity of SIRT1 and SOD2. We also found that the efficacy of ATX was closely related to the reduction of oxidative stress. As it is not clear whether the protective effect of ATX on PA adverse effects on bone mass and bone metabolism is achieved through anti-oxidative stress on osteoblasts and/or osteoclasts, we performed cell experiments to verify the changes in oxidative stress levels in MC3T3-E1 and RAW264.7 cells. In MC3T3-E1 treated with PA, MitoSOX and ROS immunofluorescence intensity increased significantly, and cell proliferation was limited, ALP expression and calcified nodule formation were inhibited, which indicated that PA treatment inhibited osteogenic differentiation by increasing the level of oxidative stress in cells. In RAW264.7 cells, PA was found to play a positive role in the induction of osteoclast differentiation, as indicated by a significant increase in TRAP formation. These results seem to imply that PA promotes bone loss in OVX rats, not only by inhibiting osteoblast activity but also by promoting osteoclast function.

When ATX is added to PA therapy, it is easy to observe that both the inhibitory effect of PA on osteogenic differentiation and the promoting effect of PA on osteoclast differentiation are disrupted by ATX. Based on the critical role of mitochondrial function in ROS production and oxidative stress [39], we therefore examined the mitochondrial membrane potential to determine the altered functional status of mitochondria. We found that PA treatment can lead to a decrease in mitochondrial membrane potential, which also indicates that PA has a damaging effect on mitochondria, while ATX can improve mitochondrial membrane potential. These results also indicated that ATX could restore the damaged mitochondria. Sirtuin 1 (SIRT1), a member of the HDAC sirtuin family, is a crucial factor in regulation of oxidative stress via its regulatory effects on mitochondrial function [40]. Therefore, we used immunofluorescence to detect the fluorescence intensity of SIRT1 to observe the oxidative stress status of cells in each group from the molecular level. Moreover, to further confirm the underlying mechanism, EX527, a specific inhibitor of SIRT1 [41], was used to investigate the functional changes in ATX/PA-treated cells. We observed that PA inhibited the expression of SIRT1 in both cells and bone tissue, while ATX could change the adverse effects of PA. In the cell experiment, ATX protected against the harmful effects of PA, while EX527 blocked the effect of ATX. These results suggest that the protective effect of ATX against PA-induced bone loss may be mediated through activation of SIRT1 signaling in OVX rats.

## Conclusion

First, this study verified that PA accelerated bone loss in osteoporosis rats by decreasing bone mass and bone mineral density and increasing oxidative stress levels in ovariectomized animal models.

In addition, ATX can reduce the level of serum oxidative stress in PA-treated rats, protect bone mass and inhibit the decrease of bone mineral density. This deleterious effect is strongly associated with accelerated osteoclast function and inhibition of osteoblast activity, resulting in bone formation being impeded. The effect of PA on the changes in the activity of osteoblasts and osteoclasts can be recovered by ATX therapy by activating SIRT1 signals. However, the lack of specific mechanism and signaling pathway studies is the main shortcoming. In addition, it is not known whether the effects of ATX and PA are directly or indirectly achieved by mediating SIRT1 signaling. Therefore, subsequent studies mainly address these doubts.

## Supplementary Material

Graphical Abstract.docx

## Data Availability

The data that support the findings of this study are available from the corresponding author [ZS Tao], upon reasonable request.
