# Supplementary material for: Astaxanthin prevents bone loss in osteoporotic rats with palmitic acid through suppressing oxidative stress
Source: Redox Rep. 2024 Apr 16;29(1):2333096. doi: 10.1080/13510002.2024.2333096 (PMC11025413; doi:10.1080/13510002.2024.2333096)
Supplement: Graphical Abstract.docx [file YRER_A_2333096_SM4897.docx]

**Graphical Abstract**


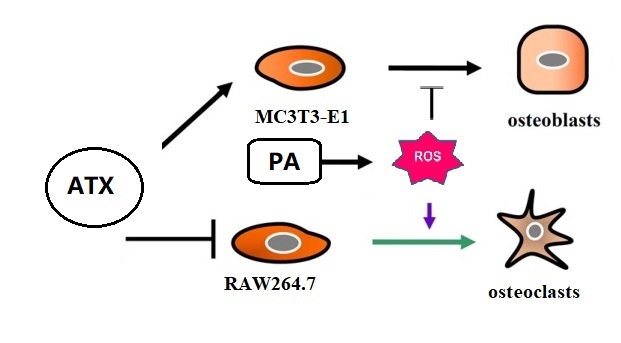


ATX may inhibit PA-induced bone loss through its antioxidant properties via the SIRT1 signaling pathway.
